# Use of fertility treatments in *BRCA1/2* mutation carriers and risk for ovarian and breast cancer: a systematic review

**DOI:** 10.1007/s00404-020-05690-4

**Published:** 2020-07-27

**Authors:** D. Huber, S. Seitz, K. Kast, G. Emons, O. Ortmann

**Affiliations:** 1 Department of Gynecology and Obstetrics, University Medical Center Regensburg, Regensburg, Germany; 2 Department of Gynecology and Obstetrics, Medical Faculty, University Hospital Carl Gustav Carus, TU Dresden, Dresden, Germany; 3 Department of Gynecology and Obstetrics, Georg August University Göttingen, University Medicine, Göttingen, Germany

**Keywords:** Fertility treatment, Fertility preservation, Breast cancer, Ovarian cancer, *BRCA1*, *BRCA2*

## Abstract

**Purpose:**

Mutations in the genes *BRCA1* and *BRCA2* represent a significant risk factor for ovarian and breast cancer. With increasing number and success rates, fertility protection and treatment are gaining importance also for *BRCA1/2* mutation carriers. However, the effect on primary cancer risk and risk for recurrence remains unclear. This review analyses the published data on fertility treatment and risk of ovarian and breast cancer in *BRCA1/2* mutation carriers.

**Methods:**

In this review, we included all relevant articles published in English from 1995 to 2018. Literature was identified through a search on PubMed and Cochrane Library.

**Results:**

We identified one retrospective cohort and one case–control study regarding the association of fertility treatments and ovarian cancer risk in *BRCA* mutation carriers. The studies show no increase in ovarian cancer risk. Furthermore, one case–control study on the association between fertility treatment and breast cancer risk in *BRCA* mutation carriers and one prospective cohort study on the long-term safety of medication used for fertility preservation in women with a history of breast cancer were identified. One of the studies shows a possible adverse effect for gonadotropin-containing medication.

**Conclusion:**

Possible increases in cancer risk associated with fertility treatments in *BRCA1/2* mutation carriers cannot be excluded at this time. Based on the existing studies, *BRCA1/2* mutation carriers should not be generally excluded from fertility treatments. However, they have to be informed about limited data and possible increases in cancer risk.

## Introduction

In the last decades, the number of fertility treatments is increasing due to postponing family planning to a later age. Improved techniques have led to enhanced success rates and social acceptance is high. A possible association between fertility treatments and ovarian cancer has been described. There are theories postulating an increasing risk promoted by polyfollicular ovulation or elevated gonadotropin levels, which are induced by fertility medication. However, the association between ovarian cancer risk and fertility treatments remains controversial [1]. A recently updated Cochrane Review presents new data suggesting that substances used in fertility treatment may cause a slight increase in ovarian cancer risk in subfertile women as compared to the general population or to subfertile women without treatment. However, none of the studies provided high-certainty evidence [2].

Large observational studies show a transient increase in breast cancer risk after the first full-term pregnancy and a long-term protective effect in uni- and biparous women as compared to nulliparous women. Higher transient breast cancer risk was observed especially in women who gave birth at an age of 30–35 years or older [3, 4]. Pregnancies terminated by spontaneous or induced abortion and do not have an impact on breast cancer risk [5]. Higher levels of estrogen and progesterone during pregnancy are discussed as underlying mechanisms that cause the transient increase in risk [3].Therefore, it has to be considered that ovarian stimulation leads to high levels of estrogen and progesterone as well.

According to a recent review published in 2017, most studies do not show an elevated risk for breast cancer in women undergoing fertility treatment as compared to parous and nonporous women without fertility treatment. However, uncertainty remains particularly when the fertility treatment is started at a young age or contains a high number of clomiphene citrate cycles [1].

Mutations in *BRCA1* and *BRCA2* genes represent a significant independent risk factor for ovarian and breast cancer. The cumulative risk to the age of 80 years for ovarian cancer is elevated up to 44% in *BRCA1* and up to 17% in *BRCA2* mutation carriers. Breast cancer risk is elevated up to 72% for *BRCA1* and up to 69% for *BRCA2* mutation carriers, respectively [6]. There are theories indicating that deficient *BRCA* function might lead to reduced fertility due to diminished ovarian reserve [7]. Similarly, more recent studies suggest a negative impact of *BRCA* mutations on fertility treatment response rates [8]. This hypothesis and the possibly occurring psychological urge to fulfill family planning in a certain time in order to schedule risk-reducing surgery procedures contribute to the fact that fertility treatment and cancer risk is of particular interest for the subgroup of *BRCA1/2* mutation carriers. This applies especially for *BRCA1/2* mutation carriers with a previous history of malignant disease. For the general population, the recent data indicate that the long-term outcome of women with pregnancy after breast cancer is not inferior to the outcome of nonpregnant matched controls [9]. This seems to be independent of hormone receptor status. The current data suggest safety for pregnancy after breast cancer also in *BRCA1/2* mutation carriers [10]. However, the data are still inhomogeneous and scarce, a higher risk for recurrence cannot be excluded. Therefore, fertility protection and fertility treatment of healthy carriers of mutations in *BRCA1* and *BRCA2* as well as of carriers after breast cancer therapy are gaining importance. In this review, we analyze the published data on fertility treatments and their association with primary ovarian and breast cancer risk and risk for recurrence in *BRCA1/2* mutation carriers.

## Materials and methods

A search for original articles was run from 1995 to 2018 on PubMed. The MeSH used for the search were: (“IVF” or “ovarian hyperstimulation”) and (“*BRCA* mutation”), and (“IVF” or “ovarian hyperstimulation”) and (“breast cancer” or “ovarian cancer”). The search created 12, 0, 99, 42, 75, and 28 hits, respectively. The hits were searched for relevance, clinical trials, reviews, and meta analyses. We also conducted a search within the Cochrane library. There were no Cochrane reviews available on fertility treatments and risk of cancer in *BRCA1/2* mutation carriers.

We found four original publications, two of which regarding ovarian cancer risk, and two regarding breast cancer risk.

## Results

### Ovarian cancer

We found one retrospective cohort study and one case–control study regarding the association of fertility treatments and ovarian cancer risk in *BRCA1/2* mutation carriers to include in our review (Table 1).

**Table 1:** Fertility treatments and risk of ovarian cancer in *BRCA* mutation carriers

Study/study design/Oxford Center of Evidence-Based Medicine (OCEBM) level of evidence (LOE)	Number	Results
Perri et al. [11] Retrospective cohort study LOE 2b	Cases: 139 *BRCA1*, 33 *BRCA2*, 3 unknown Controls: 579 *BRCA1*, 298 *BRCA2*, 3 *BRCA1*+*2*, 18 unknown	No association * BRCA1*: Any treatment: OR = 0.81; 95% CI 0.43–1.53 IVF: OR = 0.82; 95% CI 0.23–2.93 GN: OR = 0.50; 95% CI 0.06–4.26 Mixed treatments: OR = 1.8; 95% CI 0.78–4.15 * BRCA2*: Any treatment: OR = 1.01; 95% CI 0.31–3.30 IVF: OR = 1,81, 95% CI 0.20–16.48 CC: OR = 3.14, 95% CI 0.57–17.41 Mixed treatments: OR = 0.77; 95% CI 0.09–6.95
Gronwald et al. [12] Case–control study LOE 3b	Cases: 791 *BRCA1*, 150 *BRCA2* Controls: 791 *BRCA1*, 150 *BRCA2*	No association * BRCA1/2*: IVF: OR = 0.66; 95% CI 0.18–2.33, *p* = 0.52 IUI: OR = 0.83; 95% CI 0.22–3.13, *p* = 0.79 SERM: OR = 0.55; 95% CI 0.27–1.10, *p* = 0.09 GN: OR = 1.35; 95% CI 0.11–16.62, *p* = 0.82 Other: OR = 1.00 (95% CI 0.25–4.00, *p* = 1.0

Perri et al. conducted a cohort study with Jewish Israeli women undergoing genetic counseling because of family history of ovarian cancer [11]. They included a total of 718 *BRCA1* and 331 *BRCA2* mutation carriers. 139 of the *BRCA1* and 33 of the *BRCA2* mutation carriers were diagnosed with ovarian cancer. Furthermore, three healthy *BRCA1*+*2* mutation carriers and three carriers of unknown mutations with ovarian cancer as well as 18 carriers of unknown mutations without ovarian cancer were included. Among the included women, 164 reported undergoing fertility treatments. Among those were 105 *BRCA1*, 54 *BRCA2* and one *BRCA1*+*2* mutation carriers and four patients with unknown mutations. The treatments included medications containing clomiphene citrate (CC) or gonadotropins (GN) in 82 and 69 of the participants, respectively. Three patients in the CC and one in the GN group were diagnosed with ovarian cancer. 66 participants reported in vitro fertilization (IVF) and 50 received a combination of those treatments. 4 of the IVF and 10 of the mixed treatment group had ovarian cancer, respectively. The mean age of the women included was 47.1 years in the group with and 50.4 years in the group without fertility treatment. The patients with ovarian cancer had a mean age of 53.6 and the patients without ovarian cancer of 49.1 years. 13.4% of the group with and 10.1% of the group without fertility treatment had undergone prophylactic bilateral salpingo-oophorectomy (PBSO) at a mean age of 44.7 and 46.9 years in the group with and without fertility treatment, respectively. In the multivariate age-adjusted analysis, the study found no association between ovarian cancer and fertility treatments for *BRCA1* (OR = 0.81; 95% CI 0.43–1.53) and *BRCA2* mutation carriers (OR = 1.01; 95% CI 0.31–3.30). No association was found also after adjustment for the different fertility treatments, i.e., CC, GN, and IVF. Dosage, number, and duration of the different treatments were not investigated, and information on the cause of infertility was not given.

Gronwald et al. included 791 *BRCA1* and 150 *BRCA2* case–control pairs in their study. In total, 11 participants had undergone IVF and 9 intrauterine insemination (IUI) [12]. 34 had been treated with selective estrogen receptor modulators (SERM), two with gonadotropins (GN) and 8 with other fertility medications. Among the ovarian cancer cases, 4 had undergone IVF and IUI each. 12 of the ovarian cancer patients had used SERM, two GN and 4 other fertility medication. The mean age was 64 and 63 years for cases and controls. The study found no association between ovarian cancer and IVF treatment (OR = 0.66; 95% CI 0.18–2.33, *p* = 0.52) or IUI (OR = 0.83; 95% CI 0.22–3.13, *p* = 0.79) in *BRCA* mutation carriers, although the confidence intervals were wide. Likewise, no association was found between ovarian cancer and the use of fertility medication. ORs were 0.55 (95% CI 0.27–1.10, *p* = 0.09) for SERM, 1.35 (95% CI 0.11–16.62, *p* = 0.82) for GN and 1.00 (95% CI 0.25–4.00, *p* = 1.0) for other medication. Separate analyses for *BRCA1* and *BRCA2* carriers were not provided. Dosage, number, and duration of the different treatments were not examined and the cause of infertility was not provided.

### Breast cancer

We found one case–control study on the association between fertility treatment and breast cancer risk in *BRCA* mutation carriers and one prospective cohort study on the long-term safety of medication used for fertility preservation in women already diagnosed with breast cancer (Table 2).

**Table 2:** Fertility treatments and risk of breast cancer in *BRCA* mutation carriers

Study/study design/Oxford Center of Evidence-Based Medicine (OCEBM) level of evidence (LOE)	Number	Results
Kotsopoulos et al. [20] Case**–**control study LOE 3b	Cases: 1054 *BRCA1*, 326 *BRCA2* Controls: 1054 *BRCA1*, 326 *BRCA2*	No association * BRCA1*: IVF: (OR = 0.99; 95% CI 0.35–2.76I Fertility medication: OR = 1.22; 95% CI 0.76–1.94) * BRCA2*: IVF: OR = 0.88; 95% CI 0.12–6.52) Fertility medication OR = 1.25; 95% CI 0.56–2.78 * BRCA1/2*: CC: OR = 0.96; 95% CI 0.54–1.72, *p* = 0.89 Possible adverse effect * BRCA1/2*: GN: OR = 2.32; 95% CI 0.91–5.95, *p* = 0.08
Kim et. al. [14] Prospective cohort study LOE 1b	Cases (FP): 26 *BRCA1/2* Controls: 21 *BRCA1/2* In total: 28 *BRCA1*, 18 *BRCA2*, 1 *BRCA1*+*2* with breast cancer diagnosis	No difference in relapse-free or overall survival (*p* = 0.57)

Kotsopoulos et al. conducted a matched case–control study that included 1054 pairs of *BRCA1* and 326 pairs of *BRCA2* mutation carriers [13]. In total, 9 of the cases and 11 of the controls had undergone IVF. 61 of the cases and 56 of the controls reported ever use of fertility medication. Among those, 44% (24 cases and 27 controls) had used medication-containing clomiphene citrate (CC), 22% (16 cases and 10 controls) medication-containing gonadotropins (GN), and 8% other fertility medication, such as bromocriptine, a combination of various drugs or estrogen. For 26%, further information on the fertility medication was missing. The mean age was 46.0 years for cases and 46.2 years for controls. Women who had undergone bilateral mastectomy were excluded. The study did not find an association between the risk of breast cancer and IVF treatment (OR = 0.98; 95% CI = 0.39–2.45) in *BRCA1/2* mutation carriers. No association was found when adjusted for *BRCA1* (OR = 0.99; 95% CI 0.35–2.76) and *BRCA2* (OR = 0.88; 95% CI 0.12–6.52) mutation. Likewise, the study did not find an increase in risk associated with the use of fertility medication (OR = 1.21; 95% CI = 0.81–1.82) in *BRCA1/2* mutation carriers. The results were similar for *BRCA1* (OR = 1.22; 95% CI 0.76–1.94) and *BRCA2* (OR = 1.25; 95% CI 0.56–2.78) mutation carriers. These results were not significantly affected by stratification for parity. When adjusted for the type of fertility medication, the study found no association between breast cancer risk and medication-containing clomiphene citrate (OR = 0.96; 95% CI 0.54–1.72, *p* = 0.89) in *BRCA1/2* mutation carriers. A possible adverse effect was observed for GN containing fertility medication (OR = 2.32; 95% CI 0.91–5.95, *p* = 0.08). However, only few women were exposed and the effect was not statistically relevant. Dosage, number, and duration of the different treatments as well were not provided and possible effects of different causes of infertility were not examined.

Kim et al. included 337 breast cancer patients in a prospective cohort study to investigate the safety of letrozole and gonadotropin stimulation for fertility preservation (FP) [14]. 120 women underwent FP before chemotherapy, the rest served as control group. In total, the study included 28 *BRCA1*, 18 *BRCA2* carriers and one carrier of both a mutation in *BRCA1* and *BRCA2*. 26 of the mutation carriers underwent FP. The mean gonadotropin dose for ovarian stimulation was 2511.0 ± 1557.0 IU. The mean follow-up after diagnosis was 5 years in the FP group and 6.9 years for controls. The study did not find a difference in the relapse-free or overall survival between the FP and the control group using the Kaplan–Meier method (*p* = 0.57). Also, tumor size, grading, involvement of lymph nodes, hormone receptor status and HER-2/*neu* overexpression were examined. No significant effect was found for these variables when FP and control group were compared. Molecular testing was not available for all participants and numbers were low. Information on risk-reducing salpingo-oophorectomy during follow-up was not collected and separate analyses for *BRCA1* and *BRCA2* mutation carriers were not provided.

## Discussion

Studies on the cancer risk of fertility treatment in women with mutations in the genes *BRCA1* and *BRCA2* are sparse. However, the clinical need for counseling on this subject is highly relevant. In women without evidence for hereditary breast or ovarian cancer, most of the existing studies do not show an elevated risk for breast cancer after clomiphene or gonadotropin treatment [1]. Also, it is unlikely that these medications lead to increases of ovarian cancer risk.

For ovarian or breast cancer, the four available studies show no association between fertility treatments and risk of cancer in *BRCA1/2* mutation carriers. For breast cancer, one of the studies shows a possible non-significant adverse effect for gonadotropin-containing fertility medication, while another study does not confirm this effect [13, 14]. Overall, the data on the safety of fertility treatments in *BRCA* mutation carriers are limited. Most of the studies are retrospective and study populations were often small, especially regarding *BRCA2* mutation carriers. Study designs were different and only one of the studies included *BRCA1/2* mutation carriers already diagnosed with breast cancer [14]. None of the published studies investigated a possible effect of dosage, number, and duration of the different treatments. Likewise, the causes of infertility and a possible impact on cancer risk were not examined. Only one of the included studies included information on different breast cancer subtypes [14].

Prospective data from clinical registries are needed to further investigate the safety of fertility treatments in *BRCA1/2* mutation carriers and to be able to evaluate a possible impact of dosage, number, and duration of the different treatments. Longer follow-up periods are required. Counselling and treatment of families with hereditary breast and ovarian cancer under trial conditions is therefore strongly recommended. The German Consortium Hereditary Breast and Ovarian Cancer (GC-HBOC) offers all families with known or suspected hereditary risk to be registered in the HerediCaRe study, which is supported by a grant of the German Ministry of Education and Research. The prospective follow up within this registry and the international cooperations on research about genetic and non-genetic risk factors will allow a more knowledge-based view on potential risks and clinical options of prevention in the future [2, 6, 15, 16]. *BRCA1/2*-associated breast cancer differs from cancer that arises in non-carriers. In breast cancer, an onset about 20 years earlier (44 years in *BRCA1* vs 64 years for sporadic BC) and a higher number of hormone receptor negative cancers are the main differences [6, 17, 18]. And even for *BRCA1/2*-associated ovarian cancer, which is very close to sporadic ovarian cancer in terms of histopathology, the age of onset is at least 10 years earlier (59 years in *BRCA1* vs 69 years for sporadic OC) [6, 17, 18]. Exposure of lifestyle factors at different ages might influence cancer risk in carriers in a different way than expected. Until association of lifestyle and cancer risk is not fully understood, it is difficult to exclude an elevated cancer risk. Intensified research in a small, well characterized group of women such as *BRCA1/2* mutation carriers will help elucidate tumorigenesis also for the general population. An example of how evidence in the field of hereditary breast and ovarian cancer changes understanding and therapy of other cancers is the targeted therapy with PARP inhibitors [19].

Based on the existing studies, *BRCA1/2* mutation carriers should be not be excluded from utilization of fertility treatments, but at the same time take part in a clinical registry to follow up cancer history. More data will be needed since possible increases in cancer risk cannot be excluded at this time. Therefore, women with *BRCA1/2* mutations who consider fertility treatment have to be informed about the limited data. They have to be informed about possible increases in cancer risk associated with different treatments. Fertility treatments have to be used with care in this specific subgroup.
